# Toxicity of Moxifloxacin on the Growth, Photosynthesis, Antioxidant System, and Metabolism of *Microcystis aeruginosa* at Different Phosphorus Levels

**DOI:** 10.3390/toxics12080611

**Published:** 2024-08-20

**Authors:** Liang Wan, Yan Zhou, Rong Huang, Yiying Jiao, Jian Gao

**Affiliations:** 1 Key Laboratory of Intelligent Health Perception and Ecological Restoration of Rivers and Lakes, Ministry of Education, School of Civil Engineering, Architecture and Environment, Hubei University of Technology, Wuhan 430068, Chinajiaoyiying1223@163.com (Y.J.); jgao13@hotmail.com (J.G.); 2 Innovation Demonstration Base of Ecological Environment Geotechnical and Ecological Restoration of Rivers and Lakes, Hubei University of Technology, Wuhan 430068, China

**Keywords:** moxifloxacin toxicity, cyanobacteria, phosphorus, antioxidant responses, metabolomics

## Abstract

Moxifloxacin (MOX), a widely used novel antibiotic, may pose ecological risks at its actual environmental concentrations, as has been detected in aquatic systems. However, its ecotoxicity to aquatic organisms and regulatory mechanisms of phosphorus in eutrophic aqueous environments are still limited. This study aimed to analyze its physiological and biochemical parameters, including cellular growth, chlorophyll fluorescence, photosynthetic pigments, oxidative stress biomarkers, and metabolomics to elucidate the toxicity induced by environmental concentrations of MOX in *Microcystis aeruginosa* at different phosphorus levels. The results revealed that the EC_50_ values of MOX on *M. aeruginosa* at different phosphorus concentrations were 8.03, 7.84, and 6.91 μg/L, respectively, indicating MOX toxicity was exacerbated with increasing phosphorus levels. High phosphorus intensified the suppression of chlorophyll fluorescence and photosynthetic pigments, while activating the antioxidant enzyme, indicating severe peroxidation damage. Metabolomic analysis showed MOX induced different discriminating metabolites under different phosphorus levels, and perturbed more biological pathways at higher phosphorus concentrations, such as starch and sucrose metabolism, pyrimidine metabolism, and glycerolipid metabolism. This indicates that phosphorus plays an important role in regulating metabolism in *M. aeruginosa* exposed to MOX. The findings provide valuable information on the mechanisms involved in cyanobacteria responses to antibiotic stress, and offer a theoretical basis for accurately assessing antibiotic toxicity in eutrophic aqueous environments.

## 1. Introduction

Antibiotics have been extensively used since their discovery to treat both human and animal infections and as feed additives to promote animal growth [1]. However, the lack of effective control over antibiotic usage has led to a widespread environmental issue. The overall usage of antibiotics rose by 65% between 2000 and 2015 all over the world [2], consequently raising antibiotic resistance, which poses a significant threat to human health. The misuse of antibiotics is a continuing occurrence globally. For example, during the COVID-19 pandemic from 2020 to 2022, the majority of patients infected with a virus received antibiotic prescriptions even without a bacterial coinfection [3]. The majority of antibiotics used are released into the sewage drainage system as primary or raw medications through urine, feces, or directly into the environment. Only a small portion of these antibiotics are metabolized or absorbed in the body [4]. As an emerging contaminant, antibiotics can affect the growth, physiological, and biochemical functions of various aquatic organisms, posing a threat to the safety and stability of ecosystems [5].

Fluoroquinolones (FQs) are synthetic antibiotics known for their remarkable efficacy against both Gram-positive and Gram-negative bacteria and are commonly used in the treatment of various infections, such as urinary tract, respiratory tract, and skin infections [6]. These antibiotics inhibit bacterial growth and reproduction by binding to the enzyme–DNA complex and directly inhibiting DNA synthesis in bacteria [7]. Moxifloxacin (MOX, C_21_H_24_FN_3_O_4_), which is a fourth-generation fluoroquinolone that was developed for its enhanced effectiveness and bactericidal action, inhibits both DNA2-gyrase and topoisomerase IV [8]. However, the efficiency of traditional biological treatments to remove MOX is distinctly lower that of treatments used to remove other fluoroquinolones [9]. Consequently, MOX has already been detected in municipal wastewater, with concentrations ranging from 0.18 μg/L to 224 μg/L [10]. Previous studies have indicated that this antibiotic may pose significant ecological risks at its actual environmental concentrations [11,12]. However, its ecotoxicity to aquatic organisms remains limited.

*Microcystis aeruginosa* plays a vital role in water ecosystems and food chains as a primary producer. Changes in its distribution and abundance can alter community outcomes and even entire ecosystems. It is also a water bloom cyanobacterium capable of synthesizing and releasing hepatotoxic microcystins (MCs), posing a significant threat to human health and ecosystem safety. The growth of *M. aeruginosa* is highly influenced by various environmental factors, including phosphorus, nitrogen, light intensity, etc. [13]. Due to its rapid growth, high visibility, and high sensitivity to toxic substances, it has been widely employed as a model organism [14,15,16]. Much research has been conducted to investigate the effect of antibiotics such as spiramycin (SPI), tigecycline (TGC), amoxicillin (AMX), and azithromycin (AZM) on the growth, photosynthetic activity, and antioxidant system of *M. aeruginosa* [17,18]. Phosphorus, a crucial part of adenosine triphosphate (ATP), adenosine diphosphate (ADP), and nucleic acids, influences the photosynthetic phosphorylation process and the expression of photosynthesis-related genes in photosynthetic organisms [19]. Therefore, the toxicity of xenobiotics to *M. aeruginosa* can be altered by varying phosphorus concentrations in surface waters with different eutrophication conditions [15]. For instance, oxidative stress in *M. aeruginosa* can be induced by phosphorus and bisphenol A, leading to the promotion of MC production and release [20]. Additionally, the growth of *M. aeruginosa* and MC production can be stimulated by AMX, while the stress response as well as the biodegradation of AMX is upregulated by phosphorus [21]. The uptake and subsequent biotransformation of arsenate by *M. aeruginosa* are negatively regulated by phosphorus levels [22]. These studies have demonstrated that the toxicity of xenobiotics to *M. aeruginosa*, as well as the biochemical processes, can be altered by phosphorus. Phosphorus is one of the most widely spread pollutants in contaminated surface water environments. Phosphorus pollution mainly comes from mining, farming, urbanization, and industrial activities. However, the regulatory mechanisms of phosphorus have not been systematically studied, especially from the perspective of metabolomics.

The aim of this study was to reveal the toxicity induced by MOX in *M. aeruginosa* at different phosphorus levels. Thus, the physiological and biochemical parameters, including cellular growth, chlorophyll fluorescence, photosynthetic pigments, and oxidative stress biomarkers, were investigated. The metabolism of *M. aeruginosa* was also analyzed to further reveal the underlying mechanisms of MOX stress in *M. aeruginosa*. The results can provide a theoretical basis and reference for the ecological risk assessment of antibiotic residues in eutrophic aqueous environments.

## 2. Materials and Methods

### 2.1. Microalgal Strain and Culture Condition

*M. aeruginosa* (FACHB-905) was purchased from the Freshwater Algae Culture Collection, Institute of Hydrobiology (the Chinese Academy of Sciences, Wuhan, China). *M. aeruginosa* was pre-cultivated in sterilized BG11 medium [23] in a thermostatic light incubator at 25 ± 1 °C with a light intensity of 3000 lx and a light cycle of 12/12 h.

### 2.2. Antibiotic Treatment

MOX (≥99% purity) was purchased from Aladdin (Shanghai, China). The stock solution of MOX was prepared by dissolving it in deionized water at 50 mg/L and stored at 4 °C. Prior to the experiment, the stock solution was filtered through a 0.22 μm membrane for sterilization. The BG11 medium was adjusted to phosphorus concentrations of 0.2, 1, and 5 mg/L and sterilized before the experiment. Antibiotic stock solution was added during the logarithmic growth phase of *M. aeruginosa*, resulting in final MOX nominal concentrations of 0, 1, 5, and 10 μg/L. *M. aeruginosa* cultured in modulated BG11 medium without MOX served as the control group. The initial absorbance at 680 nm of *M. aeruginosa* in this experiment was approximately 0.070, with an initial algal cell count of around 3.0 × 10^6^ cells/mL. The flasks were stored under the cultivation conditions described above for 168 h, with shaking three times per day to ensure homogenous mixing. All treatments were replicated four times in this study for the purpose of metabolomics analysis.

### 2.3. Analytical Procedures

#### 2.3.1. Determination of Algal Growth

The cell density of *M. aeruginosa* was measured at 680 nm using a UV/VIS spectrophotometer (Shimadzu, Kyoto, Japan). A standard regression equation was established between absorbance values and corresponding cell counts using a spectrophotometer and hemocytometer. Three counts were performed for each sample, and the average was calculated. The regression equation *y* = 54.41*x* − 0.83 (*r^2^* = 0.99) was established and employed to compute cell counts, where *y* represents the cell density (10^6^ cells/mL) and *x* denotes OD_680_.

To evaluate the toxicity of MOX to *M. aeruginosa*, the half-effective concentrations (EC_50_) and predicted non-effect concentrations (PNECs) were calculated [24]. PNECs were calculated by dividing the EC_50_ values with assessment factor 1000 in this research [25].

#### 2.3.2. Determination of Chlorophyll Fluorescence

Chlorophyll fluorescence was determined employing a portable plant efficiency analyzer (Handy PEA, Hansatech Instruments, Norfolk, UK) [26]. The sensor unit of PEA consists of 3 ultra-bright red LEDs with a peak wavelength of 650 nm at a maximum intensity of 3500 µmol m^−2^ s^−1^. After 15 min of dark adaptation, the culture samples were measured. *Fv*/*Fm* was calculated using the formula *Fv*/*Fm* = (*Fm* − *Fo*)/*Fm*, where *Fm* represents the maximum fluorescence, and *Fo* denotes the minimum fluorescence.

#### 2.3.3. Determination of Photosynthetic Pigments

The samples were centrifuged at 8000 rpm and 4 °C for 10 min, and the supernatants were then discarded. The residual cell pellets were resuspended in 5 mL of 95% ethanol. These resuspended solutions were stored at 4 °C for 24 h for extraction, followed by centrifugation. The resulting supernatants were measured at absorbances of 665, 652, and 470 nm using a UV/VIS spectrophotometer (Shimadzu, Kyoto, Japan). The concentrations of cellular pigments were calculated using the following equations [27]:

Chlorophyll a (mg/cell) = (16.82A_665_ − 9.28A_652_)/N

Carotenoids (mg/cell) = (1000A_470_ − 1.91C_a_)/225N

where N is the cell count and C_a_ is the concentration of chlorophyll a, respectively.

#### 2.3.4. Determination of Oxidative Stress Biomarkers

The levels of reactive oxygen species (ROS) were assessed using a fluorescent staining method as described by Chen [28]. After centrifugation of the samples at 8000 rpm and 4 °C for 10 min, the supernatant was discarded, and the cell pellets were resuspended in pure water. Subsequently, the fluorescence probe 2,7-dichlorofluores-cein diacetate (DCFH-DA) was added, and the solutions were thoroughly mixed before undergoing a 30 min incubation period in darkness. A fluorescence spectrophotometer (Shimadzu, Kyoto, Japan) was employed to measure the fluorescence intensity at a fluorescence emission (Em) level of 535 nm and a fluorescence excitation (Ex) level of 524 nm. The levels of ROS were then calculated and expressed as relative to the ROS content in the control group.

The levels of malondialdehyde (MDA) were determined using the thiobarbituric acid method [29]. Following the same centrifugation protocol described for ROS, the remaining cell pellets were resuspended in a solution consisting of 2 mL of 0.6% thiobarbituric acid and 2 mL of 10% trichloroacetic acid. Subsequently, the mixed solutions were incubated in a water bath set at 100 °C for 20 min. The solutions were then centrifuged again, and the supernatants were analyzed at wavelengths of 440 nm, 532 nm, and 600 nm using a UV/VIS spectrophotometer (Shimadzu, Kyoto, Japan). The levels of cellular MDA were calculated using the following equation:

C (μmol/cell) = [6.45(A_532_ − A_600_) − 0.56A_440_]/1000N

where N is the cell count.

The cell pellets were reconstituted in precooled phosphate buffer and subjected to sonication for 5 min in an ice bath using a cell cracker. The centrifuged supernatants from the homogenate were then employed to measure the activities of antioxidant enzymes. The nitroblue tetrazolium photoreduction method was used to determine superoxide dismutase (SOD) [11]. A clear test tube was filled with reaction reagent, and it was then exposed to a 4000 lx fluorescent light for 15–20 min to develop the color. After the reaction was finished, it was stopped by covering it with a black cloth. Each test tube’s reaction solution was tested for absorbance at 560 nm using a dark control as a blank. The activities of catalase (CAT) were assessed using the UV absorption method [30]. In brief, 1 mL of extracted supernatant was combined with 2 mL of phosphate buffer and 0.3 mL of H_2_O_2_ (0.1 mol/L). The mixture was vigorously mixed and monitored using a UV/VIS spectrophotometer (Shimadzu, Kyoto, Japan) set at 240 nm. The enzyme reaction duration was 3 min, and CAT activity was determined as the decrease in OD_240_ per min.

#### 2.3.5. Metabolites Analysis Based on GC-MS

After MOX treatment at different concentrations of phosphorus for 168 h, 20 mL of *M. aeruginosa* cells was collected for metabolite analysis after centrifugation. The extraction procedure was performed according to a method described by Huang [31]. The cell pellets were washed with phosphate buffer solution (PBS) three times and then frozen in liquid nitrogen to stop metabolism. Metabolites within the cells were extracted using 1 mL of 80% methanol and 200 μL of chloroform, with 60 μL of ribitol (0.2 mg/mL) added as the internal standard. The mixtures were sonicated for 20 min to extract metabolites and then centrifuged. The supernatants were transferred to fresh tubes, and the residue mixtures underwent a repeat extraction process. The supernatants obtained from the twice extraction were dried by freeze vacuum drying. The residues were derivatized using methoxylamine hydrochloride in pyridine, followed by MSTFA. Samples were analyzed by an Agilent 7890A gas chromatography system coupled to an Agilent 5977B inert MSD system (Agilent Technologies Inc., Santa Clara, CA, USA). The method performed by GC-MS analysis followed the procedure described by Zhang [32]. Metabolites were identified and semi-quantified based on their retention index and mass spectral fingerprints using MSDIAL (v4.90) [33].

#### 2.3.6. Statistical Analyses

Each treatment was replicated four times in this research. One-way analysis of variance through the Tukey–Kramer multiple comparison test was used to evaluate significant differences. For analyzing the GC-MS data, the supervised partial least-squares discriminant analysis (PLS–DA) clustering method was utilized. The biological pathway analysis was conducted using online resources (http://www.metaboanalyst.ca/) and accessed on 16 October 2023. The impact value threshold for pathway analysis was set at 0.1.

## 3. Results and Discussion

### 3.1. Effects of MOX on Cellular Growth

**Figure 1:**
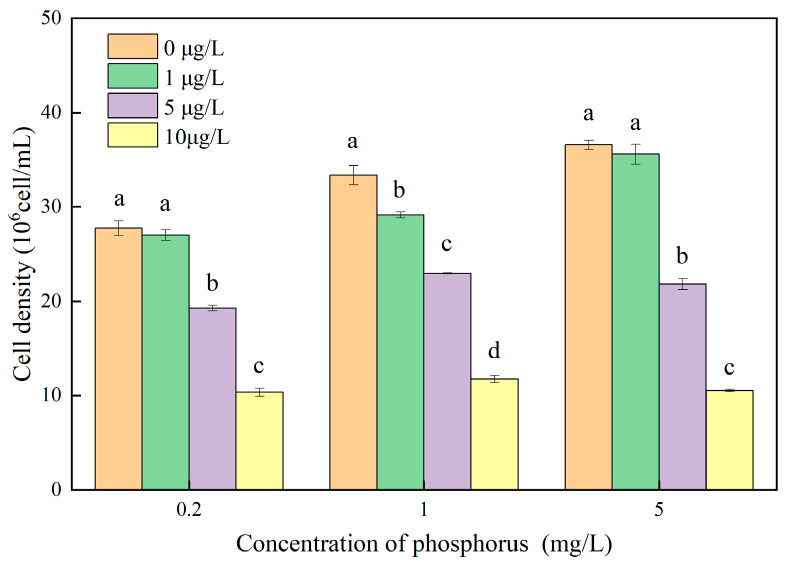
Effects of MOX on the cellular growth of *M. aeruginosa* at different phosphorus levels. Different letters indicate significant differences (*p* < 0.05) between the control and experimental at each phosphorus level (n = 4).

The impact of different concentrations of MOX on the growth of *M. aeruginosa* at various phosphorus levels is illustrated in Figure 1. The growth of *M. aeruginosa* increased with increasing phosphorus concentrations in the absence of MOX stress, indicating that phosphorus acted as the limiting factor, constraining *M. aeruginosa* growth. When *M. aeruginosa* was exposed to MOX concentrations greater than 1 μg/L, significantly reduced growth was observed. The dose–concentration–effect relationship under MOX stress was evident across all nutrient conditions. The maximum inhibition was observed at 10 μg/L MOX, with inhibition rates of 62.70, 64.80, and 71.23% at phosphorus concentrations of 0.2, 1, and 5 mg/L, respectively. The inhibition rate increased with higher phosphorus concentrations, indicating that the toxicity of MOX to *M. aeruginosa* is influenced by phosphorus conditions. To evaluate the toxicity of MOX to *M. aeruginosa* under various phosphorus concentrations, the EC_50_ and PNEC were calculated, as shown in Table 1. The EC_50_ of MOX on *M. aeruginosa* was 8.03, 7.84, and 6.91 μg/L under phosphorus concentrations of 0.2, 1, and 5 mg/L, respectively. There was no significant difference in the EC_50_ values at 0.2 and 1 mg/L phosphorus. However, the EC_50_ significantly decreased at 5 mg/L phosphorus, indicating that MOX is more toxic at higher phosphorous concentrations. The actual concentrations of MOX detected in municipal wastewater ranged from 0.18 μg/L to 224 μg/L [10], which were much higher than the PNEC concentrations, suggesting that MOX represents a high ecological risk.

The toxicity of xenobiotics to algae is generally influenced by factors such as morphology, cytology, physiology, and phylogenetics [34]. In previous studies, the 96 h EC_50_ of MOX on *M. aeruginosa* was determined to be 60.34 μg/L [11]. The higher MOX toxicity on *M. aeruginosa* in this study can be attributed to the longer exposure time. The toxicity of xenobiotics to cyanobacteria is also influenced by nutrients. For instance, phosphorus can participate in ATP synthesis, affecting the activity of H^+^-ATPase and cellular metabolism, thereby influencing algal cell growth [20]. Phosphorus, in conjunction with bisphenol A, has been found to promote the growth and photosynthesis of *M. aeruginosa*, as well as increase the synthesis and release of microcystin toxins [20]. Increased phosphorus concentrations enhance the promoting effect of AMX on the growth of *M. aeruginosa* [21]. On the contrary, high concentrations of inorganic phosphorus significantly augment the inhibition of perfluorooctanoic acid on *M. aeruginosa* [15]. These opposite results suggest that phosphorus plays a significant role in the toxicological mechanisms of *M. aeruginosa*, and the phosphorus contaminant should be fully considered in assessing the ecological risk of xenobiotics in eutrophication water.

### 3.2. Effects of MOX on Chlorophyll Fluorescence

**Figure 2:**
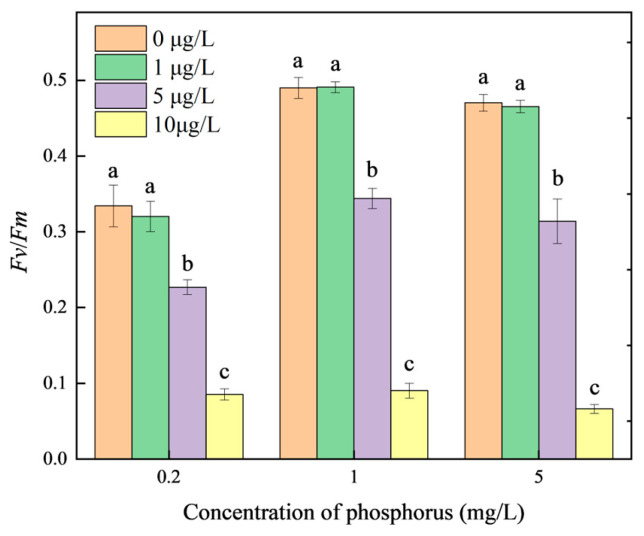
Effects of MOX on the photosynthesis of *M. aeruginosa* at different phosphorus levels. Different letters indicate significant differences (*p* < 0.05) between the control and experimental groups at each phosphorus level (n = 4).

The fluorescence parameter *Fv*/*Fm* is extensively used to represent the maximum quantum efficiency of the PSII complex in the photosystem. The impact of different concentrations of MOX on the *Fv*/*Fm* of *M. aeruginosa* at various phosphorus levels is shown in Figure 2. *Fv*/*Fm* at 0.2 mg/L phosphorus was obviously lower than at 1 and 5 mg/L without MOX stress, demonstrating that the PSII complex in *M. aeruginosa* was inhibited under phosphorus restriction. No significant changes in *Fv*/*Fm* were observed in *M. aeruginosa* between the groups exposed to 1 μg/L MOX and the control group. However, the *M. aeruginosa* groups exposed to MOX concentrations higher than 1 μg/L significantly decreased compared with the control. The observed reductions in *Fv/Fm* with 5 μg/L MOX stress were 32.09, 29.80, and 33.26% under phosphorus concentrations of 0.2, 1, and 5 mg/L, respectively, and 74.50, 81.58, and 85.92% under the corresponding phosphorus concentrations with 10 μg/L MOX stress, respectively.

The *Fv*/*Fm* index shows how susceptible *M. aeruginosa* is to changes in the external environment. Phosphorus is an essential macronutrient of thylakoid membranes, regulating the synthesis of ATP, NADPH, and phospholipids, which play pivotal roles in photosynthetic efficiency [35]. *Fv*/*Fm* in algae decreases quickly under phosphorus-limited conditions [36]. This can be attributed to the inactivation of the PSI complex and the limited extent of cyclic phosphorylation in the photosynthetic system [37]. The *psbA* gene in *M. aeruginosa*, which is related to D1 protein synthesis in PSII, was significantly upregulated by increased phosphorus concentrations, indicating the positive regulation of phosphorus on photosynthesis [21]. The photosynthetic rate and electron transfer can be quickly recovered with sufficient phosphorus after phosphorus deficiency, indicating a reversible process [37]. Additionally, the synthesis of PSII reaction centers was inhibited by the antibiotic erythromycin in *M. aeruginosa* [38]. These results further verified the regulation of phosphorus and xenobiotics on photosynthesis in *M. aeruginosa*.

### 3.3. Effects of MOX on Photosynthetic Pigments

**Figure 3:**
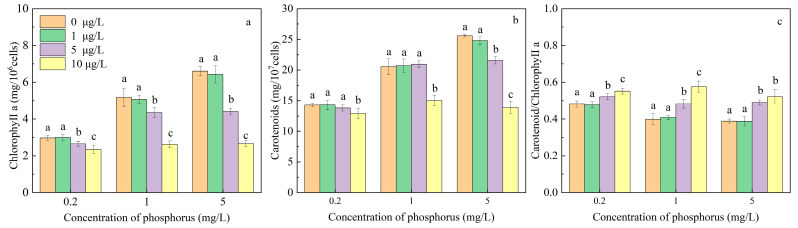
Effects of MOX on chlorophyll a content (**a**), carotenoid content (**b**), and carotenoid/chlorophyll a ratio (**c**) of *M. aeruginosa* at different phosphorus levels. Different letters indicate significant differences (*p* < 0.05) between the control and experimental at each phosphorus level (n = 4).

Chlorophyll a and carotenoids are vital photosynthetic pigments in *M. aeruginosa*, playing essential roles in energy absorption and conversion during photosynthesis. As shown in Figure 3a, the chlorophyll a content exhibited a significant increase with rising phosphorus concentrations, with no significant differences observed when comparing MOX concentrations at 1 μg/L with the control groups. However, after MOX concentrations exceeded 1 μg/L, the chlorophyll a contents significantly decreased. The highest inhibition rates of chlorophyll a content were observed to be 21.12, 49.43, and 59.64% under phosphorus concentrations of 0.2, 1, and 5 mg/L, respectively, with 10 μg/L MOX. Similarly, the carotenoid contents showed a trend similar to that of chlorophyll a, but no significant difference was observed with 5 μg/L MOX at phosphorus concentrations lower than 1 mg/L. The highest inhibition rates of carotenoid contents were 9.75, 26.75, and 45.79% under phosphorus concentrations of 0.2, 1, and 5 mg/L, respectively, with 10 μg/L MOX. The higher inhibition rate of chlorophyll a and lower inhibition rate of carotenoid contents resulted in a significant increase in the carotenoids/chlorophyll a ratio under MOX stress.

The chlorophyll a content in plants is regulated by phosphorus through a series of essential biosynthetic pathways. When phosphorus becomes deficient, the majority of photosynthates work to synthesize storage products in cells. But, with sufficient phosphorus, photosynthates are predominantly utilized by the biosynthetic pathways, leading to chlorophyll a synthesis [39]. When the synthesis rate of chlorophyll a exceeds cell division under sufficient phosphorus, the absolute content of chlorophyll a per cell increases. The chlorophyll a content in cells can also be regulated by xenobiotics. For instance, chlorophyll a content in *M. aeruginosa* increased under 17 μg/L paraquat but decreased significantly at 65 μg/L after 72 h of exposure [14]. Concentrations of norfloxacin lower than 10 μg/L did not induce a significant effect on chlorophyll a content, but 20, 30, and 50 μg/L norfloxacin significantly decreased chlorophyll a content in *M. aeruginosa* [16]. The reductions in chlorophyll a content under xenobiotic exposure are attributed to direct oxidation and breakdown by ROS induced by the interruption energy transfer in photosynthesis [40], suggesting that the breakdown of chlorophyll a acts as a protective mechanism against oxidative stress in *M. aeruginosa*.

The carotenoid contents significantly increased with increasing phosphorus concentrations in *M. aeruginosa* without MOX stress, indicating a positive correlation between carotenoid content and phosphorus levels. These results are consistent with those in other studies, for instance, phosphorus supplementation increased the carotenoid pigment contents in *Tetraselmis marina* [41]. Phosphorus has almost equal effects to nitrate in increasing carotenoids synthesis in *Asterarcys quadricellulare* [42]. Carotenoid content is also affected by xenobiotic stress. The macrolide AZM increased the content of carotenoids in *Chlorella pyrenoidosa* at concentrations lower than 1 μg/L, but decreased the synthesis of carotenoids at concentrations of more than 1 μg/L [17]. Carotenoids play a key role in the protective mechanism against oxidative from abiotic stress by quenching superoxide anions and free radicals, suppressing excited chlorophyll to alleviate lipid peroxides [14,43]. The carotenoid to chlorophyll ratio without MOX stress decreased with sufficient phosphorus and remained stable at phosphorus concentrations higher than 1 mg/L. However, this ratio significantly increased with increasing MOX concentrations at all phosphorus levels, indicating a relatively faster synthesis of carotenoids under MOX stress. This suggests that carotenoids play a more critical role in resisting peroxide damage compared to chlorophyll.

### 3.4. Effects of MOX on Oxidative Stress

**Figure 4:**
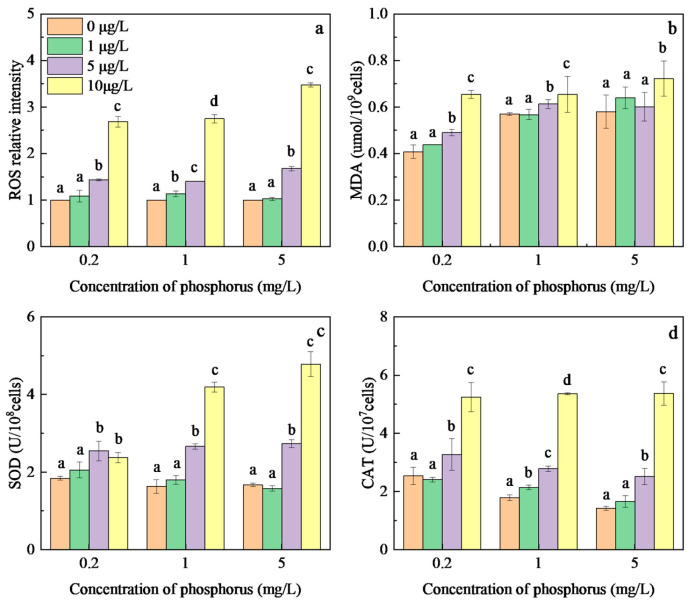
Effects of MOX on ROS activity (**a**), MDA content (**b**), SOD activity (**c**), and CAT activity (**d**) of *M. aeruginosa* at different phosphorus levels. Different letters indicate significant differences (*p* < 0.05) between the control and experimental at each phosphorus level (n = 4).

Reactive oxygen species (ROS) play an important role in regulating various biological processes in plant growth and development [44]. Excessive ROS are induced as signaling molecules under abiotic and biotic stress, enabling rapid responses to defend against different environmental stimuli [45]. The relative intensity of ROS in *M. aeruginosa* under MOX exposure at different phosphorus concentrations is shown in Figure 4a. No significant difference in ROS intensity was observed at different phosphorus concentrations under low MOX concentrations, but ROS intensity significantly increased at MOX concentrations exceeding 1 μg/L. The maximum ROS intensities were 2.68, 2.75, and 3.47 times those of the control induced by 10 μg/L MOX with phosphorus concentrations of 0.2, 1, and 5 mg/L, respectively. Xenobiotics, including MOX and azithromycin, have been shown to disrupt electron transfer within the photosystem, resulting in the excessive accumulation of ROS due to electron interactions with molecular oxygen [11,46]. Hence, peroxidation damage was induced in algal cells. Furthermore, the apparent increases in ROS intensity with increasing phosphorus concentrations under MOX stress indicate that phosphorus plays a significant role in the toxicological mechanisms of MOX on *M. aeruginosa*. Escalating ROS intensity is the primary cause of increased toxicity of MOX to *M. aeruginosa* at higher phosphorus concentrations.

MDA is one of the final products of the reaction between excessive ROS and polyunsaturated lipids in cell membranes, being frequently used as a biomarker of oxidative damage [47]. As shown in Figure 4b, the MDA contents in *M. aeruginosa* at a 0.2 mg/L phosphorus concentration were obviously lower than in the 1 and 5 mg/L groups without MOX stress. The MDA contents significantly increased with MOX exposure at all phosphorus levels, with the highest content achieved at 10 μg/L MOX, reaching 0.65, 0.65, and 0.72 μmol/10^9^ cells at phosphorus concentrations of 0.2, 1, and 5 mg/L, respectively. The significant increase in MDA content with increasing phosphorus concentrations suggests that the high-phosphorus group induced more oxidative damage than the low-phosphorus group. Furthermore, the higher increases in ROS content under MOX exposure, compared with the MDA content, suggest that the antioxidant system in *M. aeruginosa* cannot prevent the excess accumulation of ROS at high phosphorus concentrations, leading to irreversible damage to cells.

There are defense systems, including enzymatic and non-enzymatic antioxidants in cells, to scavenge excessive ROS and reduce the oxidative damage caused by environmental stresses. In this study, the enzymatic antioxidants SOD and CAT were selected to evaluate the capacities of the antioxidant system in *M. aeruginosa* against MOX exposure. The effect of the phosphorus level on SOD content after MOX exposure was consistent with the ROS content in cyanobacterial cells; that is, the low MOX concentrations at different phosphorus concentrations induced insignificant differences in SOD content, but SOD intensity significantly increased at MOX concentrations that exceeded 1 μg/L at all phosphorus concentrations. The SOD content with 10 μg/L MOX was 1.29, 2.57, and 2.87 times that of the control at phosphorus concentrations of 0.2, 1, and 5 mg/L, respectively. Similarly, another antioxidant enzyme, CAT, at the MOX concentration of 10 μg/L, significantly increased by 2.07, 3.00, and 3.77 times compared to that in the control at phosphorus concentrations of 0.2, 1, and 5 mg/L, respectively. The increase in ROS content at 0.2 mg/L phosphorus was significantly greater than that in SOD content, suggesting that the synthetic pathway of SOD is limited by phosphorus. This is because SOD acts as the first line of defense against oxidative stress by converting ROS to hydrogen peroxide and oxygen. Consistent with this research, no significant response of SOD to AMX was observed under phosphorus deficiency [21]. CAT and SOD are both essential components of the cellular antioxidant defense system, working synergistically to protect cells from oxidative damage. Hence, although the enzymatic antioxidants SOD and CAT increased significantly, ROS could not be eliminated in time, resulting in cell membrane rupture in *M. aeruginosa* cells, leading to oxidative damage and finally growth inhibition.

### 3.5. Metabolic Response of M. aeruginosa to MOX

**Figure 5:**
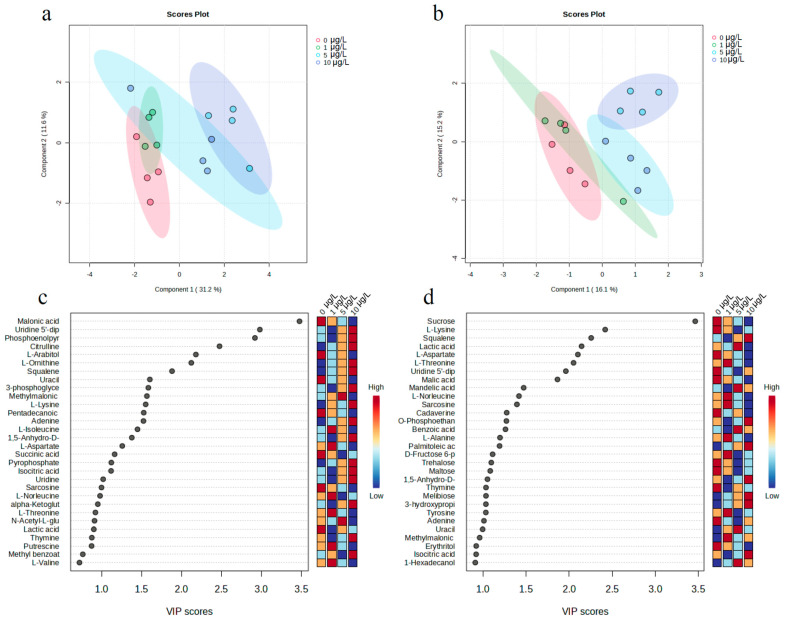
PLS-DA loading plot (**a**,**b**) and VIP score plot (**c**,**d**) of metabolites in *M. aeruginosa* exposed to MOX at 0.2 and 1 mg/L phosphorus, respectively.

The physiological response of *M. aeruginosa* to MOX exposure under different phosphorus concentrations, as discussed above, can be attributed to metabolic changes in cyanobacteria cells. To further elucidate these changes in metabolites, the groups exposed to MOX at 0.2 and 1 mg/L phosphorus were selected for investigation of metabolomic alterations, on account of the phosphorus concentrations in actual surface water. A total of 89 metabolites (Supplementary Table S1) in cyanobacteria cells were identified and semi-quantified based on the matching score according to their retention index and mass spectral fingerprints in MSDIAL [33]. Subsequently, data normalization and log transformation were conducted before analysis, and a supervised partial least-squares discriminant analysis (PLS-DA) clustering method was employed to maximize the separation between different groups and understand the class-separating information carried by variables [48]. The score plot of PLS-DA showed that the different groups were separated according to the different concentrations of MOX at both 0.2 and 1 mg/L phosphorus (Figure 5a,b), clearly indicating changes in metabolites with environmental-concentration MOX. Additionally, despite the insignificant difference in physiological responses at 1 μg/L MOX, normal metabolism was disrupted in cyanobacteria cells, as reflected by the score plot at that concentration.

The identified metabolites mainly included amino acids, organic acids, alcohols, carbohydrates, fatty acids, and others. Some of these metabolites, such as amino acids, organic acids, and carbohydrates, are known to play important roles in the stress resistance of microalgae. Generally, metabolites with variable importance in projection (VIP) scores higher than 1 were defined as potential biomarkers [49]. To screen out the metabolites contributing to the metabolic separation induced by MOX at different phosphorus concentrations, discriminating metabolites with a VIP score greater than 1 were isolated, as shown in Figure 5c,d. The results indicate that 20 and 25 metabolites out of the 89 compounds were markedly altered in response to MOX at 0.2 and 1 mg/L phosphorus, respectively. A majority of discriminating metabolites at 0.2 mg/L phosphorus, such as those involved in the cell nucleus process (such as Uridine 5′-diphosphate and Uridine), organic acids (Phosphoenolpyruvic acid), and amino acids (Citrulline and L-Ornithine), exhibited increased contents with increasing MOX concentrations. Phosphoenolpyruvic acid, which is known as a potential antioxidant and cytoprotectant that attenuates cellular injury in plants, was upregulated. This is an intermediate product of glycolysis, suggesting an antioxidant stress response of *M. aeruginosa* to MOX [50]. The increase in uridine and uridine 5′-diphosphate indicated disturbed nucleic acid synthesis in *M. aeruginosa* treated with MOX, while uridine is an intermediate in pyrimidine catabolism [51]. Additionally, the decrease in uracil suggested that MOX exposure causes alterations in RNA in cells due to the catabolic intermediates of pyrimidines [52]. Malonic acid, as a competitive inhibitor of succinic dehydrogenase, was found to be decreased, indicating the tricarboxylic acid cycle was disturbed [53]. The variation in discriminating metabolites suggests that the antioxidant system, energy production, and nucleic acid synthesis in *M. aeruginosa* were influenced by MOX at 0.2 mg/L phosphorus.

A considerable proportion of discriminating metabolites at 1 mg/L phosphorus exhibited decreased contents with increasing MOX concentrations, such as carbohydrates (e.g., sucrose, maltose, and trehalose) and amino acids (e.g., L-Lysine, L-Aspartate, and L-Threonine). The downregulation of carbohydrates indicated fluctuations in starch and sugar metabolism in *M. aeruginosa*. Carbohydrates serve as vital energy sources for growth and development, and they also act as signaling molecules for the communication of proteins, lipids, and a variety of metabolic pathways in plants [32]. It is worth noting that although trehalose metabolism is involved in the abiotic stress response in many organisms, its content decreased in this research, demonstrating that trehalose cannot be accumulated in *M. aeruginosa* to resist MOX stress [54]. Amino acids play a crucial role in modulating various physiological processes in plants. They act as osmolytes, regulate ion transport, modulate stomatal opening, and serve as precursors for synthesizing defense-related and signaling metabolites. Aspartate, a precursor of lysine through the aspartate family pathway, is involved in the biosynthesis of multiple biomolecules for plant growth and defense [55]. Lysine serves as a precursor for glutamate, an essential signaling amino acid that regulates plant growth and the response to the environment. Research suggests that lysine catabolism, through the saccharopine pathway, is highly responsive to abiotic stress [56]. The decrease in lysine likely contributed to the synthesis of fatty acids by being converted to acetyl-CoA. Threonine plays a significant role in growth and development, regulating phytohormones and inhibiting the effect of abiotic stresses such as salt, cold, and drought [57]. Additionally, threonine is involved in the synthesis of glycine, which is related to the biosynthesis of the antioxidant glutathione [58]. As aforementioned, the discriminating metabolites in different phosphorus levels were not exactly the same, indicating that the changes in metabolism in *M. aeruginosa* exposed to MOX were regulated by phosphorus.

### 3.6. Perturbed Pathway of M. aeruginosa by MOX

Biological pathway analysis was conducted based on GC-MS data, and the results are presented in Table 2. The threshold for the impact value used in identifying the biological pathway was set at 0.1. At 0.2 mg/L phosphorus, MOX perturbed four biological pathways, including cysteine and methionine metabolism; glyoxylate and dicarboxylate metabolism; lysine degradation; and alanine, aspartate, and glutamate metabolism. In contrast, MOX perturbed eight biological pathways at 1 mg/L phosphorus, including starch and sucrose metabolism; pyrimidine metabolism; arginine and proline metabolism; arginine biosynthesis; lysine degradation; glycerolipid metabolism; cysteine and methionine metabolism; and glycine, serine, and threonine metabolism. This analysis indicated that MOX induced more pronounced metabolic changes at 1 mg/L phosphorus compared to 0.2 mg/L. At 0.2 mg/L phosphorus. Three perturbed pathways were related to amino acid metabolism, suggesting that nitrogen utilization was affected by MOX, given that nitrogen is the key component of amino acid metabolism. Another perturbed pathway, glyoxylate and dicarboxylate metabolism, involves reactions related to glyoxalic acid or dicarboxylic acid. Fatty acids are degraded through oxidation and decomposition to acetyl CoA, subsequently producing malate, succinic acid, and glyoxylic acid in the tricarboxylic acid cycle [59].

**Figure 6:**
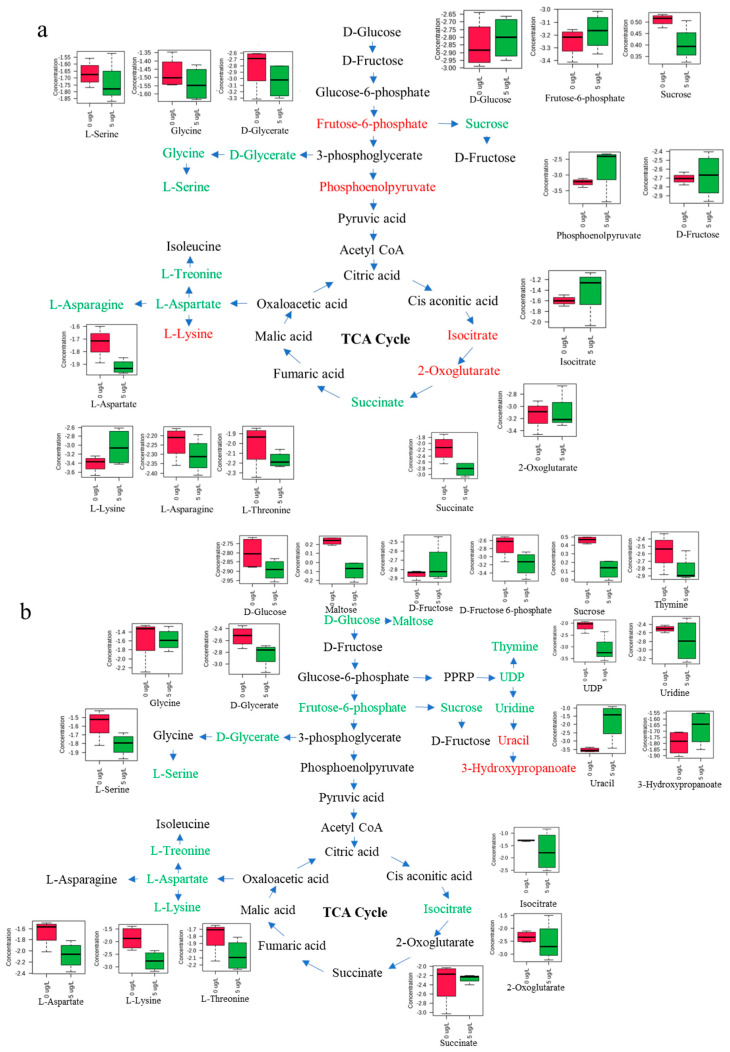
The proposed pathways disturbed in *M. aeruginosa* by MOX at 0.2 mg/L (**a**) and 1 mg/L (**b**) phosphorus. The representative metabolites encompass various metabolic pathways, including the TCA cycle and amino acid metabolism. Metabolites highlighted in red or green signify increases or decreases, respectively. Box and whisker plots depict the relative content of metabolites compared to the control group.

At 1 mg/L phosphorus, the perturbed pathways were associated with different amino acid metabolism pathways compared to those at 0.2 mg/L phosphorus, indicating that the nitrogen utilization of *M. aeruginosa* can also be influenced by phosphorus under MOX stress. Furthermore, three other biological pathways, including starch and sucrose metabolism, pyrimidine metabolism, and glycerolipid metabolism, were only perturbed at 1 mg/L phosphorus, potentially contributing to the stress resistance regulated by phosphorus. Starch and sucrose metabolism provides energy for growth and development in normal organisms and plays a crucial role in resistance to abiotic stresses. Consistent with this research, previous studies have shown that norfloxacin stress influences starch synthesis by downregulating glucose production, which is an important process in starch metabolism [60]. However, starch synthesis and lipid synthesis have a competitive relationship due to their shared precursors. This promotes the production of fatty acids while simultaneously obstructing starch synthesis under MOX stress with sufficient phosphorus. Carbon fixation could accumulate, which is insusceptible to phosphorus deficiency in photosynthetic organisms, but the cell division is inhibited [61]. Pyrimidine metabolism is crucial for providing the nucleotides necessary for nucleic acid synthesis. Under phosphorus deficiency conditions, nucleotides such as cytidine monophosphate and uridine monophosphate are significantly suppressed [62], as phosphorus is an essential component of these molecules. Additionally, pyrimidine biosynthesis can be inhibited by fluoroquinolone ciprofloxacin, leading to a compensatory increase in the uptake of pyrimidine precursors, ultimately inhibiting cell growth [63]. The glycerolipid metabolism pathway involves the synthesis of glycerolipids like monoacylglycerols, diacylglycerols, and triacylglycerols. This process is crucial as it provides cells with membranes, serves as a storage form of energy and building blocks, and produces potent signaling compounds. Based on the mentioned metabolites, schematic diagrams summarizing the proposed metabolic pathways are provided in Figure 6, including the TCA cycle, amino acid metabolism, and other metabolic pathways. In summary, fluctuations in phosphorus levels trigger a wider array of changes in metabolic pathways, notably affecting starch and sucrose metabolism, pyrimidine metabolism, and glycerolipid metabolism, all of which could be critical in *M. aeruginosa*’s response to MOX stress.

## 4. Conclusions

The present study investigated the toxicological mechanisms of environmental-concentration MOX on *M. aeruginosa* at different phosphorus levels. The physiological and biochemical parameters, including cellular growth, chlorophyll fluorescence, photosynthetic pigments, and oxidative stress biomarkers, as well as the metabolomics based on GC–MS, were systematically studied. The results showed the growth of *M. aeruginosa* was significantly inhibited by MOX and exacerbated as phosphorus concentration increased. Analysis of photosynthesis and antioxidant responses demonstrated that MOX induced peroxidation damage to *M. aeruginosa*, including the suppression of chlorophyll fluorescence and photosynthetic pigments and activation of antioxidant enzymes. Metabolomic results indicated that MOX induced different discriminating metabolites under different phosphorus levels and perturbed more biological pathways at higher phosphorus concentrations, indicating phosphorus regulation of MOX toxicity metabolism in *M. aeruginosa*. This study has provided valuable information about the mechanisms involved in cyanobacteria responses to antibiotic stress and a theoretical basis for accurately assessing antibiotic toxicity in eutrophic aqueous environments.

## Figures and Tables

**Table 1:** The EC_50_ and PNEC of MOX for *M. aeruginosa* at different phosphorus concentrations.

Concentration (mg/L)	Equation	*r* ^2^	EC_50_ (μg/L)	PNEC (ng/L)
0.2	y ^a^ = 6.42x ^b^ − 1.55	0.99	8.03	8.03
1	y = 6.61x − 1.85	0.99	7.84	7.84
5	y = 6.90x + 2.32	0.98	6.91	6.91

^a^ Specific growth rate inhibition (%). ^b^ MOX concentrations (μg/L).

**Table 2:** Perturbed pathways in *M. aeruginosa* exposed to MOX at different phosphorus concentrations.

P Concentration	Perturbed Pathways	Match Status	*p*	Impact	Involved Metabolites
0.2 mg/L	Cysteine and methionine metabolism	4/41	0.0084	0.1236	5′-Methylthioadenosine, L-Serine, L-Homoserine, L-Aspartate,
	Glyoxylate and dicarboxylate metabolism	7/37	0.0110	0.2154	Isocitrate, D-Glycerate, (S)-Malate, meso-Tartaric acid, Glycine, Succinate, L-Serine
	Lysine degradation	4/22	0.0179	0.2444	L-Lysine, Cadaverine, 2-Oxoglutarate, Succinate
	Alanine, aspartate, and glutamate metabolism	6/22	0.0210	0.2154	L-Aspartate, L-Asparagine, L-Alanine, 2-Oxoglutarate, Fumarate, Succinate
1 mg/L	Starch and sucrose metabolism	6/22	0.0124	0.5114	D-Fructose, Sucrose, D-Glucose, alpha-Trehalose, Maltose, D-Fructose 6-phosphate
	Pyrimidine metabolism	5/51	0.0120	0.2061	Uracil, Uridine, Thymine, 3-Hydroxypropanoate, Uridine 5′-diphosphate
	Arginine and proline metabolism	2/29	0.0223	0.1742	Putrescine, L-Ornithine
	Arginine biosynthesis	7/16	0.0234	0.4088	N-Acetylornithine, N-Acetyl-L-glutamate, L-Aspartate, L-Citrulline, L-Ornithine, 2-Oxoglutarate, Fumarate
	Lysine degradation	4/22	0.0300	0.2444	L-Lysine, Cadaverine, 2-Oxoglutarate, Succinate
	Glycerolipid metabolism	2/15	0.0362	0.2191	Glycerol, D-Glycerate
	Cysteine and methionine metabolism	4/41	0.0393	0.1236	5′-Methylthioadenosine, L-Serine, L-Homoserine, L-Aspartate
	Glycine, serine, and threonine metabolism	6/33	0.0473	0.5114	Glycine, L-Aspartate, L-Threonine, L-Serine, L-Homoserine, D-Glycerate

## Data Availability

The original data presented in the study are included in the article. Further inquiries can be directed to the corresponding author.

## Supplementary Materials

The following supporting information can be downloaded at: https://www.mdpi.com/article/10.3390/toxics12080611/s1, Table S1: summary of metabolites identified in MSDIAL in GC-MS.
