# Supplementary material for: Toxicity of Moxifloxacin on the Growth, Photosynthesis, Antioxidant System, and Metabolism of Microcystis aeruginosa at Different Phosphorus Levels
Source: Toxics. 2024 Aug 20;12(8):611. doi: 10.3390/toxics12080611 (PMC11359433; doi:10.3390/toxics12080611)
Supplement: Supplementary file 1 [file toxics-12-00611-s001.zip › toxics-3137624-supplementary.pdf]

Table S1: Summary of metabolites identified in MSDIAL in GC-MS.

Metabolites	RT	Molecular formula	Molecular mass	KEGG ID
Lactic acid	5.689	C3H6O3	90.08	C01432
Isopropylbenzene	6.126	C9H12	120.19	C14396
Benzonitrile	6.933	C7H5N	103.12	C09814
D-beta-Hydroxybutyric acid	6.808	C4H8O3	104.1	C01089
3-hydroxypropionic acid	6.989	C3H6O3	90.08	C01013
Sarcosine	7.338	C3H7NO2	89.09	C00213
sulfuric acid	7.404	H2SO4	98.0785	C00059
Malonic acid	8.21	C3H4O4	104.06	C00383
Methyl benzoate	8.347	C8H8O2	136.1479	C20645
L-Norleucine	10.515	C6H13NO2	131.17	C01933
4-hydroxybutyric acid	8.844	C4H8O3	104.1	C00989
L-Valine	9.049	C5H11NO2	117.15	C00183
Ethanolamine	9.297	C2H7NO	61.08	C00189
Urea	9.31	CH4N2O	60.056	C00086
Methylmalonic acid	9.217	C4H6O4	118.09	C02170
Benzoic acid	9.446	C7H6O2	122.12	C00180
Pyrophosphate	9.566	H4P2O7	177.9751	C00013
Serine	9.729	C3H7NO3	105.09	C00716
2,4-Dichloroaniline	9.529	C6H5Cl2N	162.01	C14419
L-Isoleucine	9.983	C6H13NO2	131.17	C00407
Glycerol	10.167	C3H8O3	92.09	C00116
Nicotinic acid	10.429	C6H5NO2	123.11	C00253
L-Threonine	12.603	C4H9NO3	119.12	C00188
Succinic acid	10.548	C4H6O4	118.09	C00042
Glycine	10.765	C2H5NO2	75.067	C00037
Uracil	11.294	C4H4N2O2	112.09	C00106
Glyceric acid	11.321	C3H6O4	106.08	C00258
Cyclohexylamine	11.393	C6H13N	99.1741	C00571
Fumaric acid	11.514	C4H4O4	116.07	C00122
L-Homoserine	11.724	C4H9NO3	119.12	C00263
L-Alanine	11.838	C3H7NO2	89.093	C00041
Biphenyl	12.346	C12H10	154.21	C06588
Thymine	12.83	C5H6N2O2	126.11	C00178
L-Aspartate	13.153	C4H7NO4	133.1027	C00049
Decanoic acid	13.953	C10H20O2	172.26	C01571
Mandelic acid	14.679	C8H8O3	152.15	C01984
Malic acid	15.05	C4H6O5	134.09	C00711
5-Oxoproline	15.54	C5H7NO3	129.114	C01879

Metabolites	RT	Molecular formula	Molecular mass	KEGG ID
Erythritol	15.707	$C_4H_{10}O_4$	122.12	C00503
D-Pyroglutamic acid	15.671	$C_5H_7NO_3$	129.114	C02237
Glutamate	15.702	$C_5H_9NO_4$	147.1293	C00025
alpha-Ketoglutaric acid	17.2	$C_5H_6O_5$	146.098	C00026
Phosphoenolpyruvic acid	17.887	$C_3H_5O_6P$	168.04	C00074
L-Arabitol	18.342	$C_5H_{12}O_5$	152.15	C00532
Lauric acid	18.795	$C_{12}H_{24}O_2$	200.32	C02679
meso-Tartaric acid	18.809	$C_4H_6O_6$	150.09	C00552
Putrescine	21.106	$C_4H_{12}N_2$	88.15	C00134
N-Acetyl-L-glutamate	22.082	$C_7H_{11}NO_5$	189.17	C00624
O-Phosphoethanolamine	22.609	$C_2H_8NO_4P$	141.06	C00346
3-phosphoglycerate	22.316	$C_3H_7O_7P$	186.06	C00197
L-Xylonic acid	22.454	$C_5H_{10}O_6$	166.13	C05411
D-Mannitol	22.623	$C_6H_{14}O_6$	182.172	C00392
Cadaverine	23.264	$C_5H_{14}N_2$	102.18	C01672
L-Iditol	23.286	$C_6H_{14}O_6$	182.17	C01507
L-Ornithine	23.365	$C_5H_{12}N_2O_2$	132.161	C00077
Citrulline	23.527	$C_6H_{13}N_3O_3$	175.19	C00327
Isocitric acid	23.645	$C_6H_8O_7$	192.12	C00311
Myristic acid	23.727	$C_{14}H_{28}O_2$	228.37	C06424
1,5-Anhydro-D-glucitol	23.863	$C_6H_{12}O_5$	164.16	C07326
Asparagine	24.027	$C_4H_8N_2O_3$	132.12	C16438
Adenine	24.322	$C_5H_5N_5$	135.13	C00147
Sorbose	25.195	$C_6H_{12}O_6$	180.16	C01452
Fructose	25.459	$C_6H_{12}O_6$	180.156	C01496
Glucose	25.801	$C_6H_{12}O_6$	180.16	C00031
L-Lysine	25.868	$C_6H_{14}N_2O_2$	146.19	C00047
Pentadecanoic acid	26.107	$C_{15}H_{30}O_2$	242.4	C16537
Tyramine	26.202	$C_8H_{11}NO$	137.18	C00483
Tyrosine	26.257	$C_9H_{11}NO_3$	181.19	C00082
1-Hexadecanol	26.4	$C_{16}H_{34}O$	242.44	C00823
N-Acetylornithine	27.632	$C_7H_{14}N_2O_3$	174.2	C00437
Palmitoleic acid	27.789	$C_{16}H_{30}O_2$	254.4082	C08362
Myo-Inositol	29.537	$C_6H_{12}O_6$	180.16	C00137
Phytol	31.292	$C_{20}H_{40}O$	296.53	C01389
Linoleic acid	31.967	$C_{18}H_{32}O_2$	280.45	C01595
Oleic acid	32.068	$C_{18}H_{34}O_2$	282.5	C00712
Elaidic acid	32.213	$C_{18}H_{34}O_2$	282.46	C01712
Stearic acid	32.609	$CH_3(CH_2)_{16}COOH$	284.48	C01530

Metabolites	RT	Molecular formula	Molecular mass	KEGG ID
D-Fructose 6-phosphate	34.851	C6H13O9P	260.14	C00085
Uridine	35.986	C9H12N2O6	244.2	C00299
Icosanoic acid	36.213	C20H40O2	312.5304	C06425
beta-Lactose	39.897	C12H22O11	342.29	C01970
Maltose	40.269	C12H22O11	342.3	C00208
Sucrose	40.446	C12H22O11	342.3	C00089
Trehalose	40.582	C12H22O11	342.3	C01083
5'-Deoxy-5'-(methylthio)adenosine	41.135	C11H15N5O3S	297.34	C00170
Squalene	42.114	C30H50	410.71	C00751
Melibiose	42.26	C12H22O11	342.29	C05402
Uridine 5'-diphosphate	43.242	C9H14N2O12P2	404.161	C00015
Galactinol	45.819	C12H22O11	342.29	C01235
